# The Sensitivity of Cancer Cells to Pheophorbide a-Based Photodynamic Therapy Is Enhanced by *NRF2* Silencing

**DOI:** 10.1371/journal.pone.0107158

**Published:** 2014-09-16

**Authors:** Bo-hyun Choi, In-geun Ryoo, Han Chang Kang, Mi-Kyoung Kwak

**Affiliations:** College of pharmacy, The Catholic University of Korea, Bucheon, Gyeonggi-do, Republic of Korea; MGH, MMS, United States of America

## Abstract

Photodynamic therapy (PDT) has emerged as an effective treatment for various solid tumors. The transcription factor NRF2 is known to protect against oxidative and electrophilic stress; however, its constitutive activity in cancer confers resistance to anti-cancer drugs. In the present study, we investigated NRF2 signaling as a potential molecular determinant of pheophorbide a (Pba)-based PDT by using *NRF2*-knockdown breast carcinoma MDA-MB-231 cells. Cells with stable *NRF2* knockdown showed enhanced cytotoxicity and apoptotic/necrotic cell death following PDT along with increased levels of singlet oxygen and reactive oxygen species (ROS). A confocal microscopic visualization of fluorogenic Pba demonstrated that *NRF2*-knockdown cells accumulate more Pba than control cells. A subsequent analysis of the expression of membrane drug transporters showed that the basal expression of *BCRP* is NRF2-dependent. Among measured drug transporters, the basal expression of breast cancer resistance protein (BCRP; ABCG2) was only diminished by *NRF2*-knockdown. Furthermore, after incubation with the BCRP specific inhibitor, differential cellular Pba accumulation and ROS in two cell lines were abolished. In addition, *NRF2*-knockdown cells express low level of peroxiredoxin 3 compared to the control, which implies that diminished mitochondrial ROS defense system can be contributing to PDT sensitization. The role of the NRF2-BCRP pathway in Pba-PDT response was further confirmed in colon carcinoma HT29 cells. Specifically, *NRF2* knockdown resulted in enhanced cell death and increased singlet oxygen and ROS levels following PDT through the diminished expression of BCRP. Similarly, PDT-induced ROS generation was substantially increased by treatment with *NRF2* shRNA in breast carcinoma MCF-7 cells, colon carcinoma HCT116 cells, renal carcinoma A498 cells, and glioblastoma A172 cells. Taken together, these results indicate that the manipulation of NRF2 can enhance Pba-PDT sensitivity in multiple cancer cells.

## Introduction

Photodynamic therapy (PDT) has emerged as an efficient treatment for several solid tumors [1]–[3]. PDT requires three elements: i) a photosensitizer that can be selectively targeted to tumor tissues, ii) an appropriate light source that emits low-energy and tissue-penetrating light, and iii) molecular oxygen [4]. The first step of PDT is activation of a photosensitizer by light. When the activated photosensitizer in its excited state returns to its ground state, it transfers its energy to oxygen and generates singlet oxygen (^1^O_2_), a highly reactive and short-lived reactive oxygen species (ROS), as a type II reaction. At the same time, the activated photosensitizer can react directly with cellular components and transfers a hydrogen atom forming radicals, which eventually produces oxidation products through the reaction with oxygen (type I reaction) [5]. Singlet oxygen and ROS are highly oxidizing molecules; therefore PDT-treated cells undergo cell death through both necrosis and apoptosis [6]. In addition to its direct effect on tumor cells, PDT affects the tumor's microenvironment by destroying its microvasculature and by enhancing inflammatory responses and tumor-specific immune responses [4], [7], [8].

Pheophorbide a (Pba) is a product of chlorophyll breakdown, which is isolated from silkworm excreta [9] and Chinese medicinal herb *Scutellaria barbarta* [10]. Pba absorbs light at longer wavelengths than the first-generation photosensitizer photofrin. The maximum wavelength Pba is 666 nm, whereas that of photofrin is 630 nm [11]. Because tissue penetration is enhanced by longer wavelengths, Pba has been considered as a photosensitizer for the treatment of large tumors in the peritoneal cavity [12]. Similar to photofrin, Pba induces apoptosis and necrosis in pancreatic carcinoma, leukemia, and hepatocellular carcinoma cells [13]–[15]. In uterine carcinoma cells, the underlying mechanism of apoptosis is Pba accumulation in the mitochondria, which leads to the generation of ROS and release of cytochrome c [16], [17]. Similarly, Pba causes mitochondria-dependent apoptosis in breast carcinoma MCF-7 cells [18]. Several *in vivo* animal studies have supported the efficacy of Pba-PDT in preventing tumorigenesis. For instance, a liposomal preparation of Pba-PDT delayed tumor growth in a colon carcinoma HT29 xenograft [19]. Intravenous administration of 0.3 mg/kg Pba followed by light irradiation significantly inhibited tumor growth in nude mice harboring a human hepatoma xenograft [11].

One factor determining the efficacy of PDT is the expression of ATP-binding cassette (ABC) transporters in the target tissue. These transporters control the intracellular accumulation of foreign chemicals by actively transporting them out of the cell [20]. The breast cancer resistance protein (BCRP or ABCG2) is an ABC transporter that was originally identified in doxorubicin-resistant breast cancer cells [21]. Overexpression of BCRP in tumors confers resistance to chemotherapy [22]. In addition to anti-cancer drugs, BCRP has been shown to transport porphyrin-type photosensitizers. Specifically, HEK cells overexpressing BCRP were resistant to Pba-induced cytotoxicity [23]. At the same time, *bcrp*-knockout mice were highly susceptible to dietary Pba-induced skin phototoxicity [24].

The transcription factor NF-E2-related factor 2 (NRF2) is a member of the cap’n’collar family of basic leucine-zipper (CNC-bZIP) transcription factors [25]. NRF2 activity is primarily regulated by the cytoplasmic protein Kelch-like ECH-associated protein 1 (KEAP1) [26], [27]. Through binding to the Neh2 domain of NRF2, KEAP1 mediates ubiquitinylation and subsequent proteasomal degradation of NRF2. Under conditions of oxidative and electrophilic stress, NRF2 is liberated from KEAP1 and translocates into the nucleus, resulting in the transcription of multiple target genes that encode detoxifying enzymes, antioxidant proteins, stress-response proteins, and drug transporters [28]–[30]. Because its target genes have cytoprotective effects, NRF2 is considered as a critical player in the defense system against oxidative and electrophilic stress [31]. Accordingly, several studies have shown that genetic deletion of *nrf2* is associated with increased susceptibility to tissue damage and injury resulting from environmental and endogenous stressors [28], [31], [32]. On the other hand, increasing evidence suggests that cancer cells exploit the NRF2 system for survival by adapting to the stressful tumor microenvironment [33]. NRF2 signaling is constitutively activated in several tumor types and cultured cancer cell lines, which is associated with increased tumor growth and resistance to chemotherapeutic agents. In cancer cells, NRF2 signaling is up-regulated after exposure to chemotherapeutic drugs, which confers acquired resistance to chemotherapy [34]–[36]. Similarly, PDT with hypericin in human bladder carcinoma cells resulted in elevated expression of nuclear NRF2 protein and heme oxygenase-1 (HO-1) through p38^MAPK^ and PI3K pathways [37]. Treatment of HepG2 cells with a non-toxic concentration of Pba followed by photo activation for 90 min resulted in increased expression of BCRP and heme oxygenase-1 (HO-1) in a NRF2-dependent manner [38].

In the present study, we investigated NRF2 as a novel molecular determinant of PDT efficacy. Because NRF2 regulates the expression of ROS-counteracting components and several drugs transporters, we hypothesized that manipulating NRF2 expression would enhance the efficacy of PDT. To test this hypothesis, we established stable *NRF2*-knockdown cell lines using human breast carcinoma MDA-MB-231 cells and colon carcinoma HT29 cells, and measured PDT sensitivity. Our results showed that *NRF2* knockdown enhances PDT-induced cell death by increasing the production of ROS. As an underlying mechanism, BCRP expression was repressed by *NRF2* knockdown, leading to increased cellular accumulation of Pba and increased production of singlet oxygen.

## Materials and Methods

### Materials

Pba was from Santa Cruz Biotechnology (Santa Cruz, CA, USA). NRF2 antibody was obtained from Abcam (Cambridge, MA, USA). Antibodies recognizing AKR1C1 and BCRP were purchased from Abnova (Taipei City, Taiwan) and Cell Signaling Technology (Beverly, MA, USA), respectively. PRDX3 and β-tubulin antibodies were obtained from Santa Cruz Biotechnology. Ko143, 3-(4,5-dimethylthiazol-2-yl)-2,5-diphenyltetrazolium bromide (MTT), and puromycin were obtained from Sigma-Aldrich (Saint Louis, MO, USA). 5(6)-Carboxy-2′,7′-dichlorofluorescein diacetate (DCFDA), trans-1-(2′methoxyvinyl) pyrene, and MitoGreen were purchased from Life Technologies (Carlsbad, CA, USA). The lentiviral system containing a pre-designed human *NRF2* shRNA and nonspecific scrambled RNA (scRNA) was bought from Sigma-Aldrich.

### Cell culture

The human breast cancer cell line MDA-MB-231 and MCF-7, colon cancer cell line HT29 and HCT116, and human renal carcinoma A498 were obtained from American Type Culture Collection (Rockville, MD, USA). The human glioblastoma cell line A172 was purchased from Korean Cell Line Bank (Seoul, South Korea). MDA-MB-231, MCF7, A498, A172, and HCT116 were maintained in a medium containing Dulbecco's Modified Eagle Medium and Nutrient Mixture F-12 (Hyclone, Utah, USA) in the ratio of 1∶1 supplemented with 10% fetal bovine serum (Hyclone) and penicillin/streptomycin (WelGene Inc., Daegu, South Korea). HT29 was maintained in RPMI-1640 medium (Hyclone) with 10% FBS and penicillin/streptomycin. The cells were grown at 37°C in a humidified 5% CO_2_ atmosphere.

### Production of lentiviral particles containing *NRF2* shRNA expression plasmid

Lentiviral particles with shRNA were produced in HEK 293T cells following the transfection of cells with *NRF2* shRNA expression plasmid and the packaging mix (Sigma-Aldrich) as described previously [36]. Briefly, HEK 293T cells were seeded in 60 mm plates at a density of 7×10^5^ cells per well. The next day, medium was replaced by OptiMEM (Life Technologies) and subsequently, 1.5 µg pLKO.1-NRF2 shRNA, which contains the human *NRF2*-specific shRNA (5′-CCGGGCTCCTACTGTGATGTGAAATCTCGAGATTTCACATCACAGTAGGA-3′), and the packaging mix were transfected into cells using Lipofectamine 2000 (Life Technologies). The pLKO.1-scRNA plasmid was used as a nonspecific control RNA. On the second day, after the removal of transfection complex, the complete medium was added into each well. Media containing lentiviral particles were harvested after 4 days.

### Establishment of *NRF* knockdown cell line

MDA-MB-231 cells in 6-well plate were incubated with lentiviral particles containing either scRNA or NRF2 shRNA expression plasmid. After a 48 h-incubation, cells were recovered in the complete medium and the puromycin (1 µg/ml) selection was followed for up to 4 weeks. The *NRF2* knockdown HT29 cell line was established as previously reported [39].

### Transient knockdown of NRF2

MCF-7, HCT116, A172 and A498 cells were seeded in 6-well plates at a density of 1×10^5^ cells/well and grown for overnight. Next day, the cells were incubated with cholesterol for 15 min and then, the lentiviral particle containing scRNA or shNRF2 were added to each well. After 48 h incubation, viral particle-containing media were removed and cells were recovered in the fresh medium for overnight.

### PDT and MTT analysis

MDA-MB-231 and HT29 were seeded at a density of 7×10^3^ cells/well in 96-well plates and incubated for 20 h. The cells were treated with Pba (0–2.5 µg/ml) for 6 h and then irradiated with 0.3 (HT29) or 0.6 J/cm^2^ (MDA-MB-231) laser in the absence of Pba. For the laser irradiation, the 670 nm LED Hybrid Lamp system (Quantum Spectra Life, Barneveld, WI) was used. The cells were recovered in complete medium for 18 h and incubated with MTT for 4 h. After removal of the MTT solution, 100 µl of dimethyl sulfoxide (DMSO) was added to each well and the absorbance was measured at 570 nm using a Spectro-star Nano microplate reader (BMG Labtechnologies, Offenburg, Germany).

### Cytotoxicity measurement

Cytotoxicity by PDT was assessed using CytoTox-Fluor assay system (Promega, Madison, WI, USA). After PDT, cells were maintained for 24 h and then 50 µl bis-AAF-R110 substrate was added to each well. Substrate containing solution was incubated for a further 2 h with orbital shaking at 37°C. Intensities of fluorescence were measured using a SpectraMax M5 (Molecular Devices, Sunnyvale, CA) at the 485 nm Ex/520 nm Em.

### Total RNA extraction and real-time PCR analysis

Total RNAs were isolated from cells using a Trizol reagent (Life Technologies). For the synthesis of cDNAs, reverse transcriptase (RT) reactions were performed by incubating 200 ng of total RNA with a reaction mixture containing 0.5 µg/µl oligo dT_12–18_ and 200 U/µl Moloney Murine Leukemia Virus RT (Life Technologies). For the real-time polymerase-chain reaction (PCR) analysis, Roche LightCycler (Mannheim, Germany) was used with the Takara SYBR Premix ExTaq system (Otsu, Japan). Primers were synthesized by Bioneer (Daejeon, South Korea) and the primer sequences for the human genes are: *NRF2*, 5′-ATAGCTGAGCCCAGTATC-3′ and 5′-CATGCACGTGAGTGCTCT-3′; *HO-1,* 5′-GCTGCTGACCCATGACACCAAGG-3′ and 5′-AAGGACCCATCGGAGAAGCG-GAG-3′; aldo-keto reductase 1C1 (*AKR1C1),* 5′-CGAGAAGAACCATGGGTGGA-3′ and 5′-GGCCACAAA-GGACTGGGTCC-3′; NAD(P) quinone oxidoreductase-1 (*NQO1*), 5′- CAGTGGTTTGGAGTCCCTGCC-3′ and 5′-TCCCCGTGGATCCCTTGCAG-3′; the catalytic subunits of γ-glutamate cysteine ligase (*GCLC*), 5′-TGAAGGGACACCAGGACAGCC-3′ and 5′-GCAGTGTGAACCCAGGACAGC-3′; epoxide hydrolase-1 (*EPHX1*), 5′-GCCTGCACTTGAACATGGCT-3′ and 5′-ATGTGCATGTAGCCGCTCTC-3′; superoxide dismutase 1 (*SOD1*), 5′-GATTCCATGTTCATGAGTTT-3′ and 5′-AGGATAACAGATGAGTTAAG-3′; *SOD2*, 5′-AACCTCACATCAACGCGCAGAT-3′and 5′-TCAGTGCAGGCTGAAGAGCTAT-3′; catalase (*CAT*), 5′- GTGCATGCAGGACAATCAGG-3′ and 5′-GAATGCCCGCACCTGAGTAA-3′; peroxiredoxin 3 (*PRDX3*), 5′- GCCGTTGTCAATGGAGAGTTC-3′ and 5′-GCAAGATGGCTAAAGTGGGAA-3′; *PRDX5*, 5′- GGTGGCCTGTCTGAGTGTTA-3′ and 5′- ACCACCATGGAGAACCTCTTG-3′; glutathione peroxidase 1 (*GPX1*), 5′-TTCCCGTGCAACCAGTTTG-3′ and 5′-TTCACCTCGCACTTCTCGAA-3′; *GPX4*, 5′-TCACCAAGTTCCTCATCGACA-3′ and 5′- GCCACACACTTGTGGAGCTA-3′; glutathione reductase (*GSR*), 5′-ACCCCGATGTATCACGCAGTTA-3′ and 5′-TGTCAAAGTCTGCCTTCGTTGC-3′; glutaredoxin 2 (*GLRX2*), 5′-GTATTGCTCTCCATCCTCCTCG-3′ and 5′- CTGGGAGCCTTTATGAGCGT-3′. thioredoxin-2 (*TXN2*), 5′-CACACCACTGTGCGTGGAAA-3′ and 5′-ACTGTAACACCCAACCCAGC-3′; breast cancer resistance protein (*BCRP/ABCG2*), 5′- CACAACCATTGCATCTTGGCTG-3′ and 5′- TGAGAGATCGATGCCCTGCTTT-3′; multidrug resistance protein 1 (*MDR1/ABCB1*), 5′-CTATGCTGGATGTTTCCGGT-3′ and 5′-TCTTCACCTGGCTCAGT-3′; multidrug resistance-associated protein 1 (*MRP1/ABCC1*), 5′-AGCTTTATGCCTGGGAGCTGGC-3′ and 5′-CGGCAAATGTG- CACAAGGCCAC-3′; *MRP2/ABCC2,* 5′-GCTGCCACACTTCAGGCTCT-3′ and 5′-GGCAGCCAGCAGTGAAAAGC-3′; *MRP3/ABCC3,* 5′-ATACGCTCGCCACAGT-CCTT-3′ and 5′-GCTGGCCATGATGACCACAA-3′; *MRP4/ABCC4*, 5′-CTTG- GATCGCAA-TACCCTTG-3′ and 5′-GACACCTCTCTTCTGCTTTG-3′; *MRP5/ABCC5,* 5′-CAGAGACCGTGAAGATTCCA-3′ and 5′-TTTGGAAGTAGTCCGGATGG-3′; *MRP6/ABCC6,* 5′-TGTGTGGCTCACCACGATG-3′ and 5′-CATAGGTAG- GTGGACAG-GTGG-3′; organic cation transporter novel 1 (*OCTN1;* SLC22A4), 5′-GCTGTATGTCTTCACTGCTG-3′ and 5′-GGTGAGGATTCCAATCAGGA-3′; *OCTN2* (*SLC22A5*), 5′-ATTGTTGTGCCTTCCACTATC-3′ and 5′-GGTCATCCACAGCATTATGG-3′; multidrug and toxin extrusion transporter 2 (*MATE2*; *SLC47A2*), 5′-TTCATTCCAGGACTTCCGGTG-3′ and 5′-AGGTGTGAGTGAGATGGATGG-3′; hypoxanthine phosphoribosyltransferase-1 (HPRT1), 5′-TGGCGTCGTGATTAGTGATG-3′ and 5′-GCTACAATGTG-ATGGCCTCC-3′.

### Measurement of singlet oxygen

The cells were seeded in a cover glass dish (SPL Life science, Gyeonggi-do, South Korea) and cultured for overnight. After the Pba-laser irradiation, the cells were washed with PBS and incubated with 50 µM trans-1-(2′methoxyvinyl) pyrene (Life Technologies) for 30 min. For the nuclei staining, Hoechst 33342 (H342) was added to the above dishes and incubated for 10 min. Green fluorescence from singlet oxygen was detected immediately using a LSM 710 confocal microscope (Carl Zeiss, Jena, Germany) and intensities were quantified using a ZEN2011 software (Carl Zeiss).

### Measurement of intracellular ROS

Cellular ROS levels were examined using a cell-permeable fluorogenic probe, carboxy-H_2_DCFDA. The cells were seeded in a cover glass bottom dish (SPL Life science) and further incubated for overnight. After the Pba-laser therapy, cells were washed with PBS and incubated with 30 µM of carboxy-H_2_DCFDA for 30 min at 37°C. For the nuclei staining, H342 was incubated for 10 min. Then confocal images were obtained and green fluorescent intensities were quantified using a LSM 710 confocal microscope and ZEN2011 software.

### Measurement of Pba accumulation

Pba is a fluorogenic substance; therefore the level of intracellular accumulation of Pba was determined by measuring red fluorescence in the cell. MDA-MB-231 and HT29 on cover glass slides were incubated with Pba for 6 h. Then, Pba was removed and PBS washes were followed. Intensities of Pba red fluorescence were quantified in confocal images using ZEN2011 software. As an alternative way for quantification, cells were seeded in 96-well plates (BD Biosciences) and incubated with Pba for 6 h. After the cell wash, Pba red fluorescence was detected using a CellInsight Personal Cell Imager (Thermo Fisher Scientific, Waltham, MA, USA) and intensities were quantified by CellInsight software (Thermo Fisher Scientific).

### Fluorometric determination of intracellular Pba

Cells in 96-well plates were incubated with Pba for 6 h and then were lysed using 1% SDS after PBS wash. DMSO was added to cell lysates to dissolve cellular Pba and fluorescence intensity was measured using a SpectraMax M5 at the 415 nm Ex/673 nm Em.

### Western blot analysis

Cells were lysed with RIPA buffer (50 mM Tris [pH 7.4], 150 mM NaCl, 1 mM EDTA, and 1% nonyl phenoxypolyethoxylethanol 40) containing a protease inhibitor cocktail (Sigma-Aldrich). For BCRP protein extraction, 0.1% SDS was added to RIPA buffer. The protein concentration was determined using a DC protein assay kit (Bio-Rad, Hercules, CA, USA). The protein samples were separated by electrophoresis on 12% SDS-polyacrylamide gels and transferred to nitrocellulose membranes (Whatman, Dassel, Germany). Then, the membrane was blocked with 5% skim milk or 3% bovine serum albumin for 1 h and incubated with antibodies. The chemiluminescence images were captured using a Fujifilm LAS-4000 mini imager (Fujifilm, Tokyo, Japan) and intensities were quantified with corresponding software.

### Flow cytometry

The NRFi-MDA cells were seeded in 60 cm^2^ dishes at a density of 1×10^5^ cells/well and cultured overnight. After the Pba-laser irradiation, the cells were trypsinized and spun down at 15,000 rpm for 10 min at 4°C. Then, 2×10^5^ cells were transferred to 1.5 ml tube for centrifugation at 8,000 *g* for 5 min at 4°C. Then, 5 µl of fluorochrome conjugated Annexin V (BioLegend, San Diego, CA, USA) and 10 µl of propidium iodide (PI, BioLegend) solution were added into each tube and incubated with gentle vortex. The cells were incubated for 15 min at room temperature in the dark and then, 400 ul of Annexin V binding buffer (BioLegend) was added to each tube. Stained cells were analyzed using a Becton-Dickinson FACS Canto (SanJose, CA, USA) with and data were analyzed with FACSDiva software (BD).

### Statistical analysis

Statistical significance was determined by a Student paired t-test followed or a one-way analysis of variance (one-way ANOVA) followed by the Student Newman–Keuls test for multiple comparisons using GraphPad Prism software (La Jolla CA, USA).

## Results

### Pba cytotoxicity is enhanced by *NRF2* knockdown in MDA-MB-231 breast cancer cells

**Figure 1:**
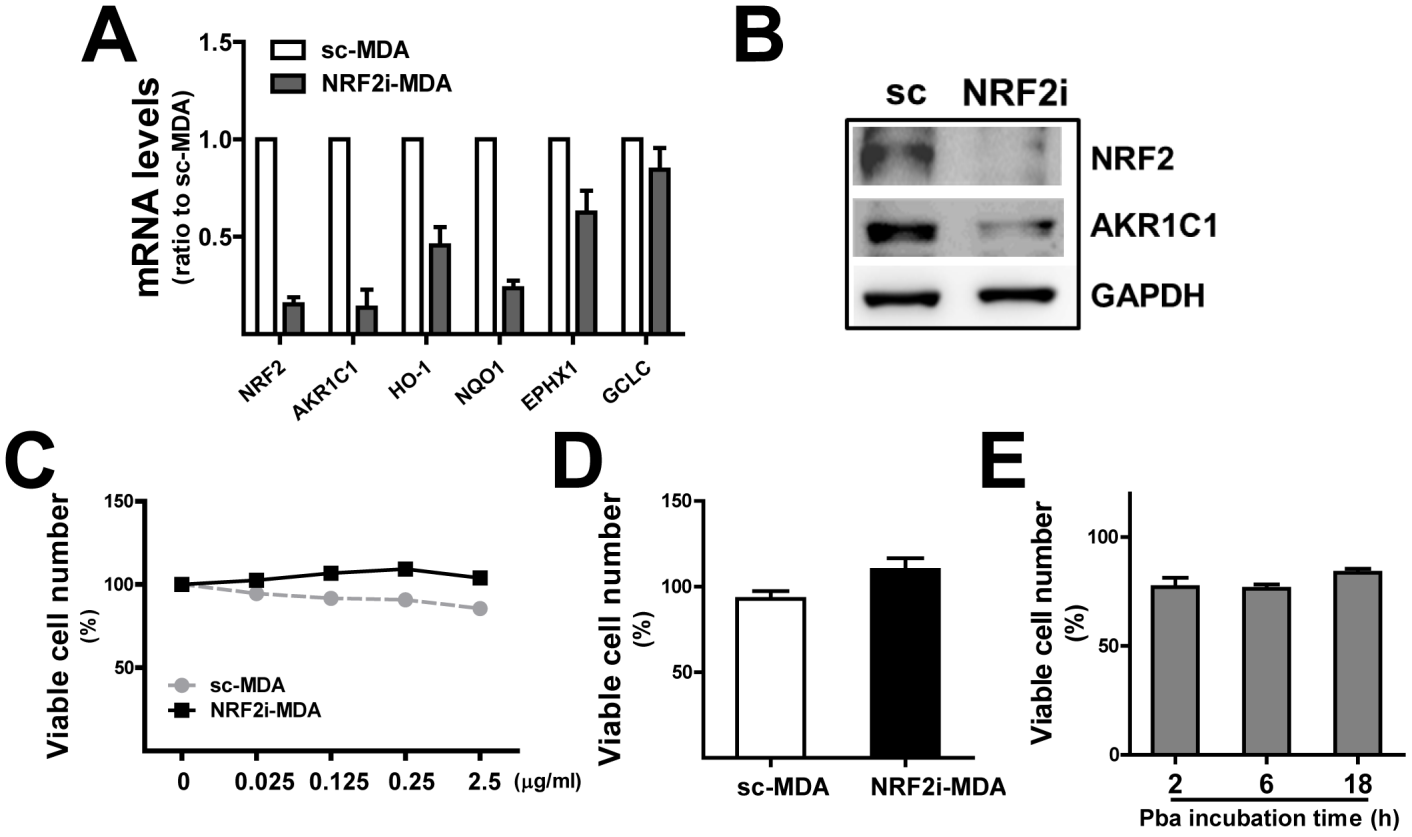
Effect of Pba and laser irradiation on MDA-MB-231. (A) Transcript levels of *NRF2*, *AKR1C1*, and *HO-1* were measured in the control (sc-MDA) and *NRF2*-knockdown cells (NRF2i-MDA) using relative quantification of real-time PCR. HPRT1 was used as a housekeeping control gene. Data represent ratios with respect to sc-MDA and are reported as mean ± standard deviations (SDs) of 3 experiments. (B) Protein levels of NRF2 and AKR1C1 were determined by Western blot analysis in the sc control and NRF2i-MDA cells. (C) The sc-MDA and NRF2i-MDA cells were incubated with Pba (0–2.5 µg/mL) for 6 h, and viable cells were quantified using an MTT assay after an 18 h recovery. (D) Cells were irradiated by a laser in the absence of Pba, and viable cells were quantified using an MTT assay after an 18 h recovery. (E) The sc-MDA was incubated with Pba for 2, 6, or 18 h and viable cell number was determined after laser irradiation. The data represent percentages with respect to the vehicle group for each cell line and are reported as the mean ±SD of 8 wells.

In order to investigate the involvement of NRF2 in the efficacy of Pba-PDT for breast cancer, we established an *NRF2*-knockdown breast carcinoma cell line in MDA- MB-231 cells. Stable cell lines expressing nonspecific scrambled RNA (sc-MDA) or *NRF2*-specific shRNA (NRF2i-MDA) were attained from puromycin selection for 4 weeks. The expression levels of NRF2 and its target genes were evaluated in these cell lines. NRF2i-MDA cells showed an 85% reduction in *NRF2* mRNA and substantial decreases in expression of the NRF2 targets *AKR1C1* and *HO-1* (Fig. 1A). Western blot analysis showed that proteins levels of NRF2 and AKR1C are substantially diminished in knockdown cells (Fig. 1B). These confirm the successful inhibition of NRF2 signaling in established stable cell line. Next, we examined the sensitivity of *NRF2*-knockdown MDA-MB-231 cells to the Pba and laser combination therapy. Without laser irradiation, incubation of sc-MDA and NRF2i-MDA cells with Pba (0.025–2.5 µg/mL, 24 h) did not significantly affect cell viability (Fig. 1C). Similarly, laser irradiation without Pba incubation did not affect cell viability (Fig. 1D). To assess PDT sensitivity, cells were irradiated with a 0.6-J/cm^2^ laser after incubation with Pba and MTT analysis was performed following 18 h recovery. Initially, PDT effect was evaluated with varied incubation times of Pba. The incubation of Pba (0.125 µg/mL) for 2, 6, and 18 h showed similar cytotoxicity in the sc control cells (Fig. 1E). Based on this result, the 6 h-incubation of Pba was maintained in this study.

**Figure 2:**
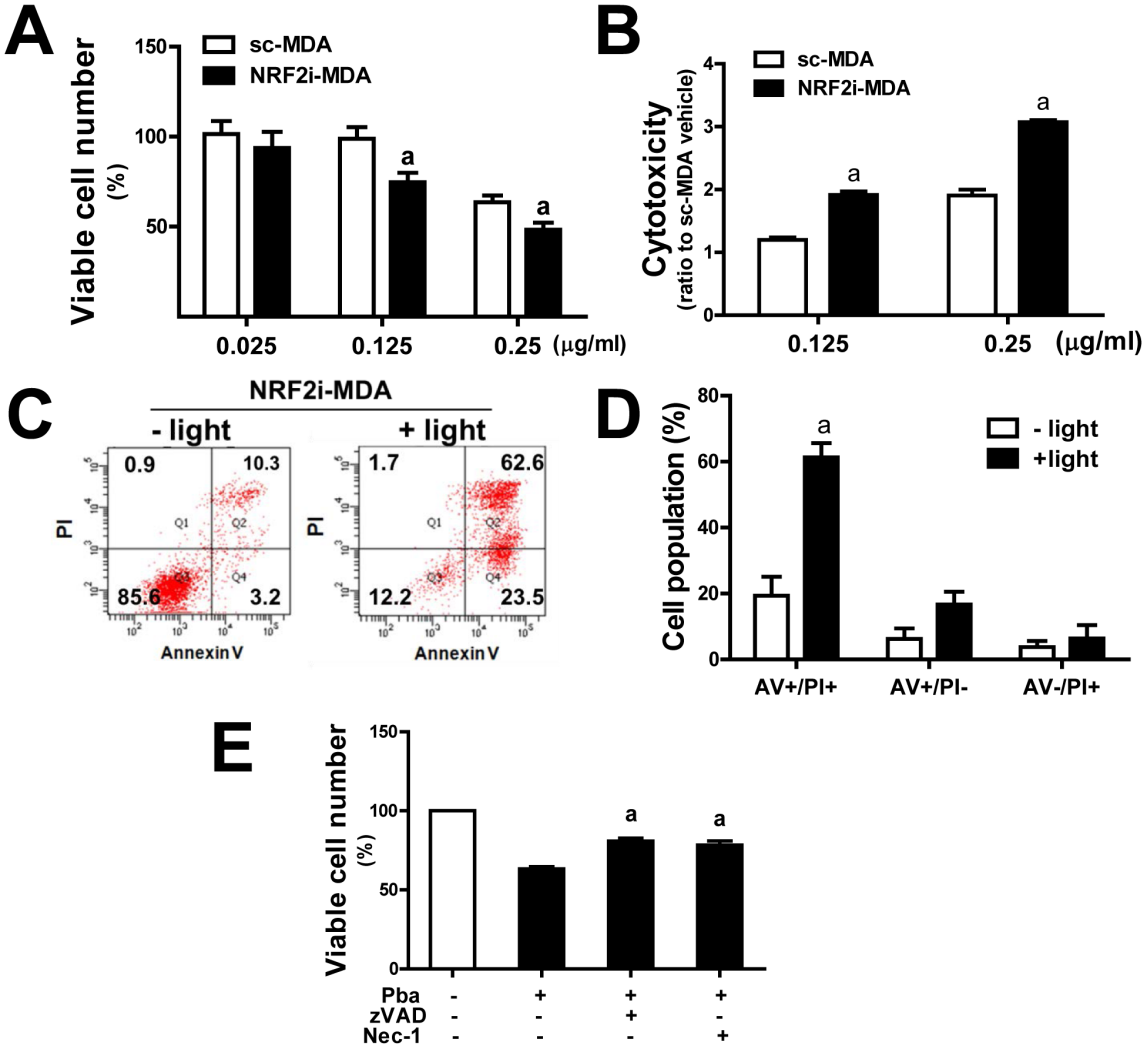
Effect of *NRF2* knockdown on Pba-laser treatment-induced cytotoxicity in MDA-MB-231. (A) The sc control and NRF2i-MDA cells were pre-incubated with Pba (0–2.5 µg/mL) for 6 h and were then irradiated by 0.6 J/cm^2^ laser. Then MTT analysis was performed following an 18 h recovery. The data represent percentages with respect to the vehicle group for each cell line and are reported as the mean ±SD of 8 wells. ^a^P<0.05 as compared with the sc-MDA control at each concentration of Pba. (B) PDT-induced cytotoxicity was assessed by measuring dead cell protease activity in a cell cultured medium. Cells were incubated with Pba (0.125 and 0.25 µg/mL) for 6 h followed by laser irradiation, and incubated for 18 h. Dead cell derived protease activity was determined using fluorogenic substrate AAF-R110. The data represent relative cytotoxicity with respect to the vehicle group for each cell line and are reported as the mean ±SD of 8 wells. ^a^P<0.05 as compared with each sc-MDA control. (C) A flow cytometric analysis of apoptotic and necrotic cells was performed in NRF2i-MDA cells after Pba-laser treatment. The cells were incubated with PI and Annexin V, and cell populations were assessed. (D) The bar graph represents cell population in Annexin V (AV)+/PI+, AV+/PI-, or AV-/PI+ phase from three separate experiments. ^a^P<0.05 as compared with no laser control. (E) NRF2i-MDA cells were co-incubated with Z-VAD-FMK (10 µM) or necrostatin (Nec-1, 50 µM) and Pba (0.125 µg/mL) for 6 h; then, cells were washed with PBS and irradiated with a laser. After 18 h incubation, viable cells were quantified using an MTT assay. The data are percentages with respect to the control (no PDT and vehicle treatment) and are reported as the mean ±SD of 8 wells. ^a^P<0.05 as compared with the control.

In MTT analysis, the NRF2-MDA cells were relatively more sensitive to Pba-based PDT: viable cell number after PDT was lower in the NRF2i-MDA cells than that in the control cells (Fig. 2A). Similarly, when dead cell-derived peptidase activity was measured in the medium using a CytoTox substrate *NRF2* knockdown cells showed a significantly high cytotoxicity compared to the sc control (Fig. 2B).

PDT induces cell death through apoptotic and necrotic pathways [6], [40]. Next, in order to identify the mechanism of cell death involved in PDT-treated *NRF2* knockdown breast cancer cells, FACS analysis with Annexin V and PI double staining was performed. NRF2i-MDA cells were treated with PDT; immediately thereafter, cells were trypsinized and stained with Annexin V (early apoptosis marker) and PI (late apoptosis and necrosis marker). After Pba-laser combination treatment, the majority cell population shifted to the apoptotic-necrotic phase (Fig. 2C). Quantification of experimental repeats showed that 16.7% of cells were in the early apoptotic phase (Annexin V+/PI-) and 61.3% of cells were in the late apoptotic/necrotic phase (Annexin V+/PI+). Only 15.7% of cells were in the non-apoptotic and non-necrotic phase (Fig. 2D). These clearly show that PDT induces cell death involving the process of apoptosis and necrosis. As a confirmation, when cells were co-incubated with a caspase pan-inhibitor Z-VAD-FMK (10 µM) and PDT (Pba 0.125 µg/mL) percentage of viable cell number increased from 63% to 80.1% (Fig. 2E). In addition, the incubation with necrostatin (50 µM), a potent inhibitor of programed necrotic cell death, elevated viable cell number to 78.2%. Taken together, obtained results indicate that *NRF2* knockdown sensitized breast cancer MDA-MB-231 cells to Pba-based PDT, resulting in enhanced cell death.

### PDT-stimulated ROS generation is elevated in *NRF2* knockdown MDA cells

**Figure 3:**
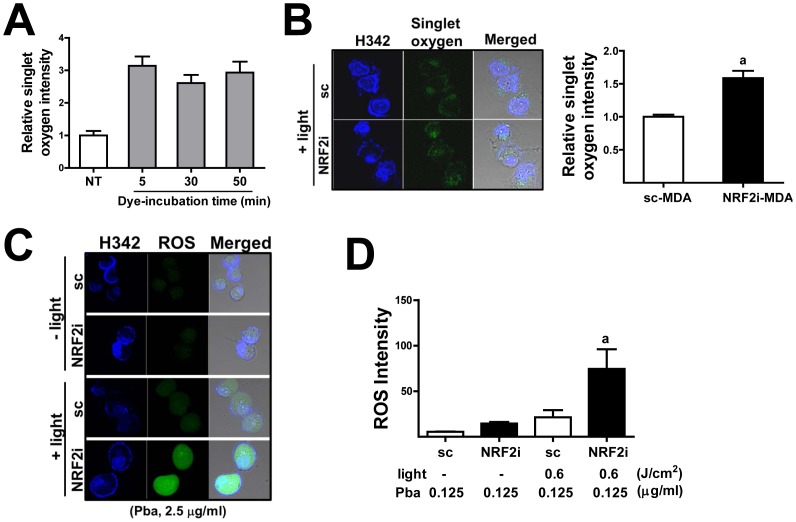
Singlet oxygen and ROS are elevated in *NRF2*-knockdown MDA-MB-231 cells. (A) The sc control cells were incubated with Pba for 6 h and irradiated by a 0.6 J/cm^2^ laser. Right after PDT, a singlet oxygen sensitive trans-1-(2′methoxyvinyl) pyrene was added and cells were further incubated for 5, 30, or 50 min in the presence of trans-1-(2′methoxyvinyl) pyrene. Then confocal observation was performed to detect fluorogenic dye formation, which was formed by the reaction with singlet oxygen. (B) The sc and NRF2i cells were incubated with trans-1-(2′methoxyvinyl) pyrene for 30 min following PDT. Then confocal microscopic detection of singlet oxygen-derived fluorogenic pyrene was carried out. Green fluorescence from singlet oxygen reacted pyrene was quantified using a ZEN 2011 software. The data represent ratios with respect to sc-MDA cells and are reported as the mean ±SD of 3 experiments. ^a^ *P*<0.05 as compared with the sc-MDA control. Hoechst 33342 (H342) presents nuclear staining. ×100 magnification (C–D) The sc- and NRF2i-MDA cells were incubated with 2.5 µg/mL (C) or 0.125 µg/mL Pba (D) for 6 h and irradiated by a 0.6 J/cm^2^ laser. Then, cells were incubated with DCFHA for 30 min, and ROS-derived green fluorescence was detected using a confocal microscope. The data represent ratios with respect to sc-MDA control cells treated with Pba only and are reported as the mean ±SD of 3 experiments. ^a^ *P*<0.05 as compared with sc-MDA with Pba and laser irradiation. ×100 magnification.

Pba-laser combination therapy releases singlet oxygen within the cell, and the resulting ROS is a major contributor to PDT's cytotoxicity [5]. Therefore, we monitored PDT-derived singlet oxygen levels in both cell lines. Cells pre-treated with Pba (2.5 µg/mL for 6 h) were irradiated by a 0.6-J/cm^2^ laser, and the levels of singlet oxygen were determined using trans-1-(2′methoxyvinyl) pyrene, a fluorescent dye, which is specific to singlet oxygen. Compared to treatment with Pba only or laser irradiation only, the trans-1-(2′methoxyvinyl) pyrene-based method showed that Pba-laser combination group displays an enhanced fluorescent signal within the cell (data not shown), confirming the increase in singlet oxygen upon PDT. In our initial assessment, the incubation of trans-1-(2′methoxyvinyl) pyrene for 5 min, 30 min and 50 min showed similar increase levels of singlet oxygen-derived fluorogenic pyrene intensity (Fig. 3A). This confirms that singlet oxygen-derived pyrene fluorescence can be reliably detectable during these incubations times; therefore a confocal determination was done after 30 min incubation of trans-1-(2′methoxyvinyl) pyrene in our study. The comparative measurement of singlet oxygen demonstrated that *NRF2*-knockdown MDA-MB-231 cells generate higher levels of singlet oxygen than the control cells (Fig. 3B). The fluorescent signals were distributed within the cytoplasm as well as the nucleus, and the total fluorescence intensity was 1.5-fold higher in the knockdown cells than that in the control cells. Accordingly, the total level of ROS, which was monitored using DCFDA, was substantially elevated after treatment with the Pba-laser combination in both cell lines; however, the increase was greater in the NRF2i-MDA cells. Specifically, after treatment with 2.5 µg/mL Pba and laser irradiation, the increase in ROS was 4.6-fold higher in the *NRF2*-knockdown cell line than in the control cell line (Fig. 3C). Similarly, after treatment with the low concentration of Pba (0.125 µg/mL) and laser irradiation, the increase in ROS was 3.5-fold higher in NRF2i-MDA cells (Fig. 3D). These results suggested that elevation in singlet oxygen and ROS enhances the sensitivity of *NRF2*-knockdown breast cancer cells to PDT.

### Increased accumulation of Pba in *NRF2* knockdown breast cancer cells is resulted from diminished BCRP expression

**Figure 4:**
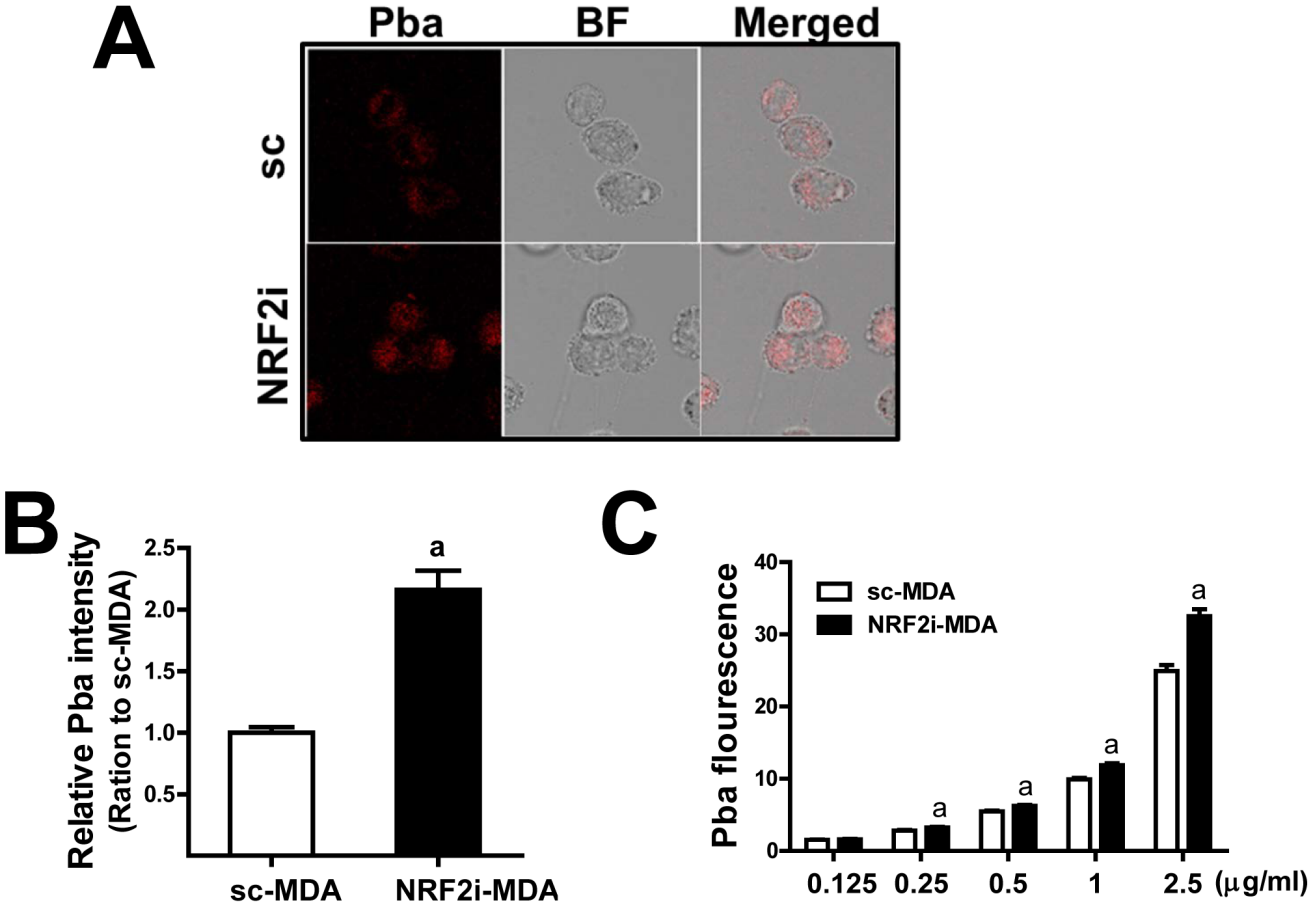
*NRF2* knockdown increases cellular accumulation of Pba. (A) The cells were incubated with Pba (2.5 µg/mL) for 6 h and then washed with PBS. Intracellular accumulation of Pba was monitored by confocal microscopy. BF, bright field; ×100 magnification (B) Intracellular accumulation of Pba (2.5 µg/mL, 6 h) was quantified using a Cell Insight system. The data are ratios with respect to the sc-MDA control and are reported as the mean ± standard error (SE) of 6 experiments. (C) Intracellular accumulation of Pba (0.25 and 0.5 µg/mL, 6 h) was assessed by fluorometric measurement in cell lysates. The data represent ratios with respect to each no PDT control are reported as the mean ±SD of 3 experiments. ^a^ *P*<0.05 as compared with sc-MDA with PDT.

The level of PDT-derived singlet oxygen was higher in NRF2i-MDA cells than in control cells (Fig. 3B), suggesting differential cellular level of Pba in *NRF2*-knockdown cells. Using the fluorescent property of Pba, we used confocal microscopy to monitor cellular levels of Pba (2.5 µg/ml) after a 6 h-incubation. The Pba-derived red fluorescence signal was greater in NRF2i-MDA cells than in control cells (Fig. 4A). Compared to control cells, NRF2i-MDA cells showed 2.2-fold greater Pba-derived fluorescent intensity when quantified using a Cell Insight System (Fig. 4B). The fluorometric measurement of Pba in cell lysates also showed similar results: cellular level of Pba is higher in knockdown cells throughout Pba concentrations (Fig. 4C).

**Figure 5:**
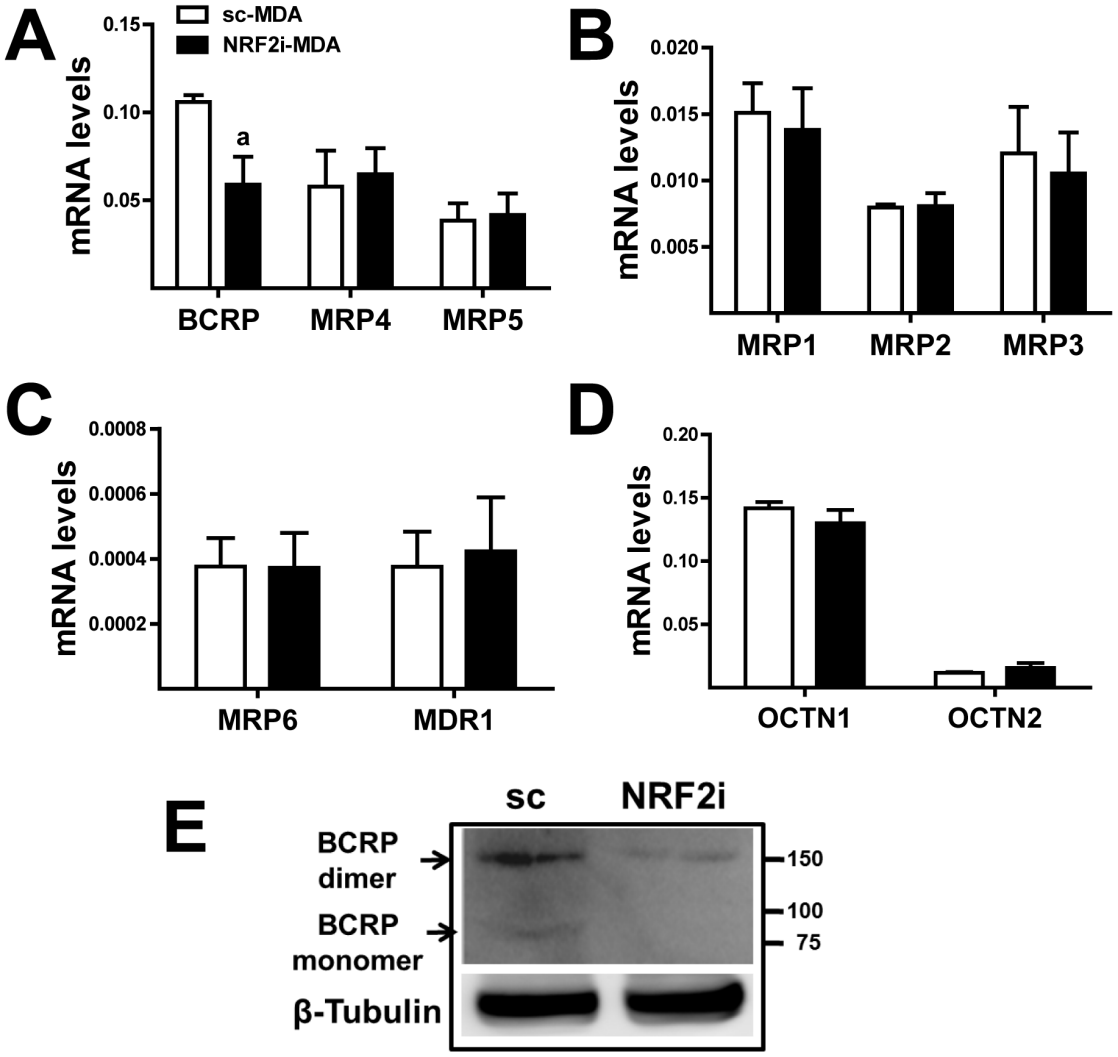
Increased Pba accumulation in *NRF2* knockdown cells is resulted from BCRP decrease. (A–C) Relative quantification of mRNA levels of ABC transporters (BCRP, MRP1-6, and MDR1) was performed in sc-DMA and NRF2i-MDA cells using real-time PCR. (D) Transcript levels of SLC transporters (OCTN1 and OCTN2) were determined using real-time PCR. The data are ratios with respect to the sc-MDA control for each gene and are reported as the mean ±SD of 3 experiments. (E) BCRP protein levels (dimer, 150 kDa; monomer, 75 kDa) in sc-MDA and NRF2i-MDA cells were determined using immunoblot analysis.

These results demonstrated that NRF2 affects cellular accumulation of Pba and that differential expression of transporters may be associated with enhanced accumulation. Next, we measured mRNA levels of several ATP-binding cassette (ABC) transporters (*BCRP*, *MRP1-6*, and *MDR1*) and solute carrier (SLC) transporters (*OCTN1* and *OCTN2*) in order to identify the mechanisms underlying Pba accumulation in NRF2-MDA cells. Among these transporters, the transcript level of *BCRP* was significantly lower in NRF2i-MAD cells than that in control cells, whereas the levels of other transporters were not altered by *NRF2* knockdown (Fig. 5A–5D). The reduced level of BCRP in *NRF2* knockdown cells was confirmed by western-blot analysis: NRF2i-MDA cells displayed significantly reduced levels of the BCRP monomers and dimers (Fig. 5E). These data indicated that the basal expression of BCRP is controlled by NRF2 in MDA-MB-231 cells and that enhanced Pba accumulation in *NRF2*-knockdown cells can be associated with reduced expression of BCRP.

### BCRP inhibitor treatment increases Pba accumulation, PDT-induced ROS increase and cytotoxicity

**Figure 6:**
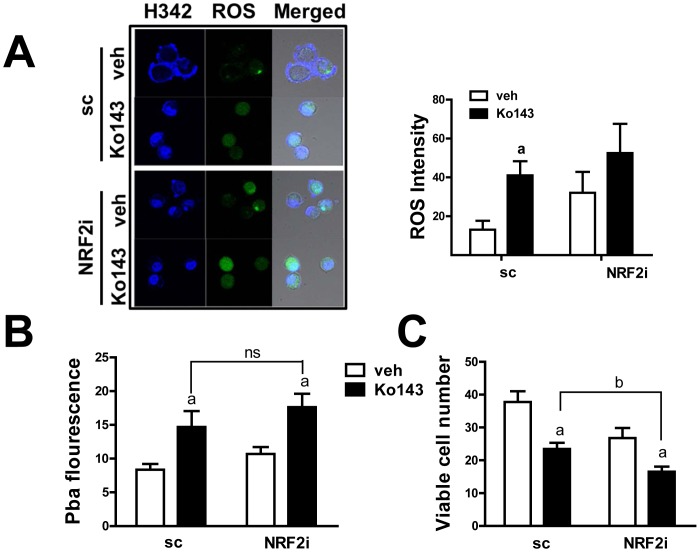
BCRP inhibitor treatment reverses Pba accumulation and PDT cytotoxicity in NRF2 knockdown cells. (A) The sc- and NRF2i-MDA cells were pre-incubated with the BCRP inhibitor Ko143 (0.5 µM) for 1 h followed by incubation with Pba (0.25 µg/mL) for 6 h. Then, the cells were irradiated with a 0.6 J/cm^2^ laser, and ROS levels were quantified by DCFDA staining. The data are ratios with respect to the sc-MDA control without Ko146 pre-incubation and are reported as the mean ±SD of 3 experiments. (B–C) After PDT in the presence of Ko143, cellular level of Pba (B) and cell viability (C) were assessed. Cellular Pba level was determined by fluorometeric measurement of Pba in cell lysates. Cell viability was measured using MTT analysis. The data are reported as the mean ±SD of 8–10 wells. ^a^ *P*<0.05 as compared with no Ko143 group of each cell line. ^b^ *P*<0.05 as compared with the vehicle-treated sc (B) or Ko143-treated sc control group (C).

In order to strengthen the relationship between BRCP and PDT sensitivity, we incubated cells with a potent BCRP inhibitor, Ko143, and monitored PDT-derived ROS. The differential increase in ROS after PDT treatment between sc-MDA and NRF2i-MDA cells disappeared when they were co-incubated with 0.5 µM Ko143. Specifically, ROS generation in sc-MDA cells increased to the same level as that in NRF2i-MDA cells (Fig. 6A). Similarly, differential cellular levels of Pba in the sc control and NRF2 knockdown cells were abolished by the Ko143 incubation (Fig. 6B). In addition, viable cell number was significantly decreased by BCRP inhibitor (Fig. 6C). These results further support the role of NRF2-BCRP in Pba-PDT response. It is notable that the effects of Ko143 on Pba accumulation and cell viability are not identical. Upon Ko143 incubation, there was no statistical difference in cellular Pba levels in both cell lines, implying the critical role of BCRP in Pba accumulation; however the viability of knockdown cells was still low compared to the sc control. These suggest the role of additional NRF2 factors in PDT response determination.

### The mitochondrial antioxidant peroxiredoxin 3 (PRDX3) is reduced in *NRF2* knockdown MDA-MB-231 cells

**Figure 7:**
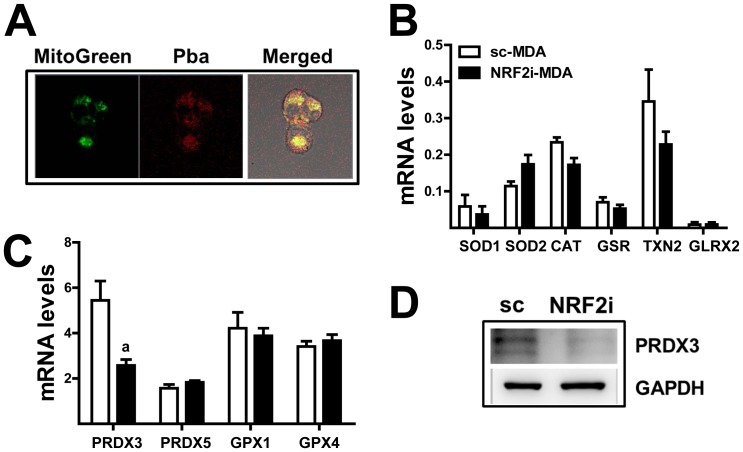
The mitochondrial ROS-counteracting protein PRDX3 is decreased in NRF2i-DMA cells. (A) The sc-MDA cells were incubated with 2.5 µg/mL Pba for 6 h and then washed with PBS. The cells were incubated with MitoGreen (0.5 µM) for 15 min, and a confocal microscopic observation was performed. Green fluorescence indicates mitochondria, red fluorescence indicates cellular Pba, and yellow color indicates co-localization of mitochondria and Pba. ×100 magnification (B–C) Transcript levels of ROS-scavenging genes were measured using real-time PCR analysis for relative quantification. The data are reported as the mean ±SD of 3–4 experiments. ^a^ *P*<0.05 as compared with the sc-MDA control for each gene. (D) Protein level of PRDX3 in the sc- and NRF2i- MDA was assessed using Western blot analysis.

In an attempt to identify additional NRF2 factors participating PDT sensitization, the levels of mitochondrial ROS counteracting enzymes were examined. Particularly, it has been reported that PDT-induced cell death is associated with the accumulation of Pba in the mitochondria [17]. When cellular Pba localization was monitored in MDA-MB-231 cells after Pba incubation, Pba-derived red fluorescence and mitochondria-specific green fluorescence were largely co-localized (Fig. 7A). These data support the Pba accumulated in the mitochondria of MDA-MB-231 cells; therefore, the mitochondrial antioxidant system can be an additional determinant of PDT sensitivity. Among the 11 mitochondrial antioxidant genes we measured, only *PRDX3* was significantly down-regulated (∼50%) by *NRF2* knockdown as compared to the control (Fig. 7B and 7C). The transcript levels of other genes, including superoxide dismutase 1/2 (SOD1/2), catalase (CAT), thioredoxin 2 (TXN2), glutaredoxin 1/4 (GLRX1/4), and PRDX5, were not affected by *NRF2* knockdown. The decrease of PRDX3 was confirmed in Western blot analysis (Fig. 7D). These results suggested that the basal expression of *PRDX3* is dependent on NRF2 and that decreased expression of this major mitochondrial ROS defense gene can participate in enhanced PDT response of knockdown cells.

### PDT sensitivity is enhanced by *NRF2* knockdown in colon cancer HT29 cells

**Figure 8:**
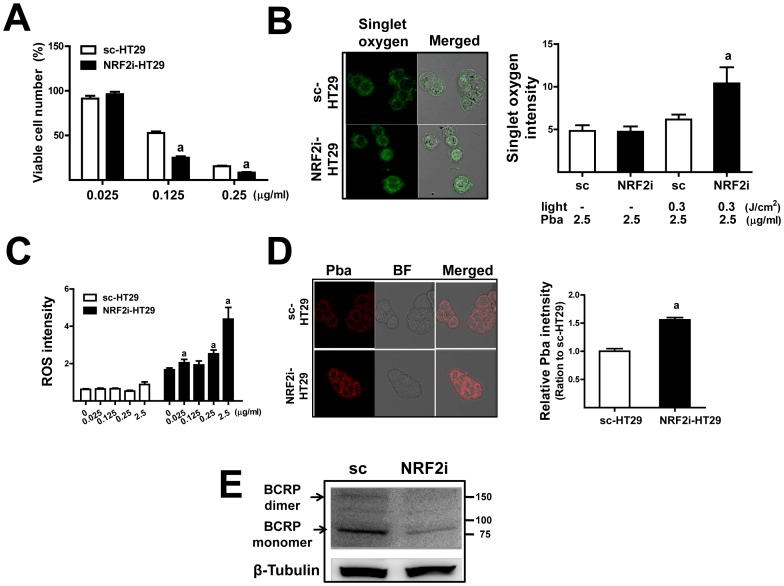
NRF2-BCRP inhibition enhances PDT sensitivity in colon cancer HT29 cells. (A) The HT29 cell lines with stable expression of nonspecific scRNA (sc-HT) and *NRF2* shRNA (NRF2i-HT) were incubated with Pba (0.025–2.5 µg/mL) for 6 h and then irradiated by a 0.3 J/cm^2^ laser. After 18 h incubation in complete medium, viable cells were quantified using an MTT assay. The data are ratios with respect to the vehicle-treated sc-HT cells and are reported as the mean ±SD of 8 wells. ^a^ *P*<0.05 as compared with each sc-HT PDT group. (B) Singlet oxygen levels were quantified in PDT-treated sc- and NRF2i-HT cells using a specific fluorescent dye. The data are reported as the mean ±SD of 3 experiments. ^a^ *P*<0.05 as compared with the sc-HT PDT group. ×100 magnification (C) Levels of ROS in PDT-treated cells were quantified using a Cell Insight Personal Cell Imager. ^a^ *P*<0.05 as compared with the no PDT group of each cell line. (D) The cells were incubated with Pba (2.5 µg/mL) for 6 h, and cellular Pba accumulation was monitored using a confocal microscopy. Pba accumulation was quantified using a Cell Insight system. The data are ratios with respect to the sc-HT control and are reported as the mean ±SE of 6 samples. BF, bright field; ×100 magnification (E) BCRP levels in sc-HT and NRF2i-HT cells were assessed by immunoblot analysis.

In order to confirm the role of NRF2-BCRP signaling in regulating the sensitivity to PDT, we examined the effect of *NRF2* knockdown on PDT response in the colon carcinoma HT29 cell line. The stable *NRF2-*knockdown cell line (NRF2i-HT) showed a substantial reduction in *NRF2*, *AKR1C1*, and *HO-1* mRNA (data not shown). MTT analysis following Pba and laser irradiation (0.3 J/cm^2^) showed that *NRF2-*knockdown HT29 cells were more sensitive to PDT-induced cytotoxicity (Fig. 8A). Furthermore, the levels of singlet oxygen and ROS were higher in NRF2i-HT cells than in control cells (Fig. 8B and 8C). Finally, similar to MDA-MB-231 cells, there was greater intracellular Pba accumulation and lower expression of *BCRP* in NRF2i-HT cells (Fig. 8D and 8E). Taken together, these results indicated that NRF2 inhibition in colon cancer cells reduced *BCRP* expression and thereby enhanced PDT sensitivity.

### Combination of lentiviral shRNA delivery and PDT in other types of cancer

**Figure 9:**
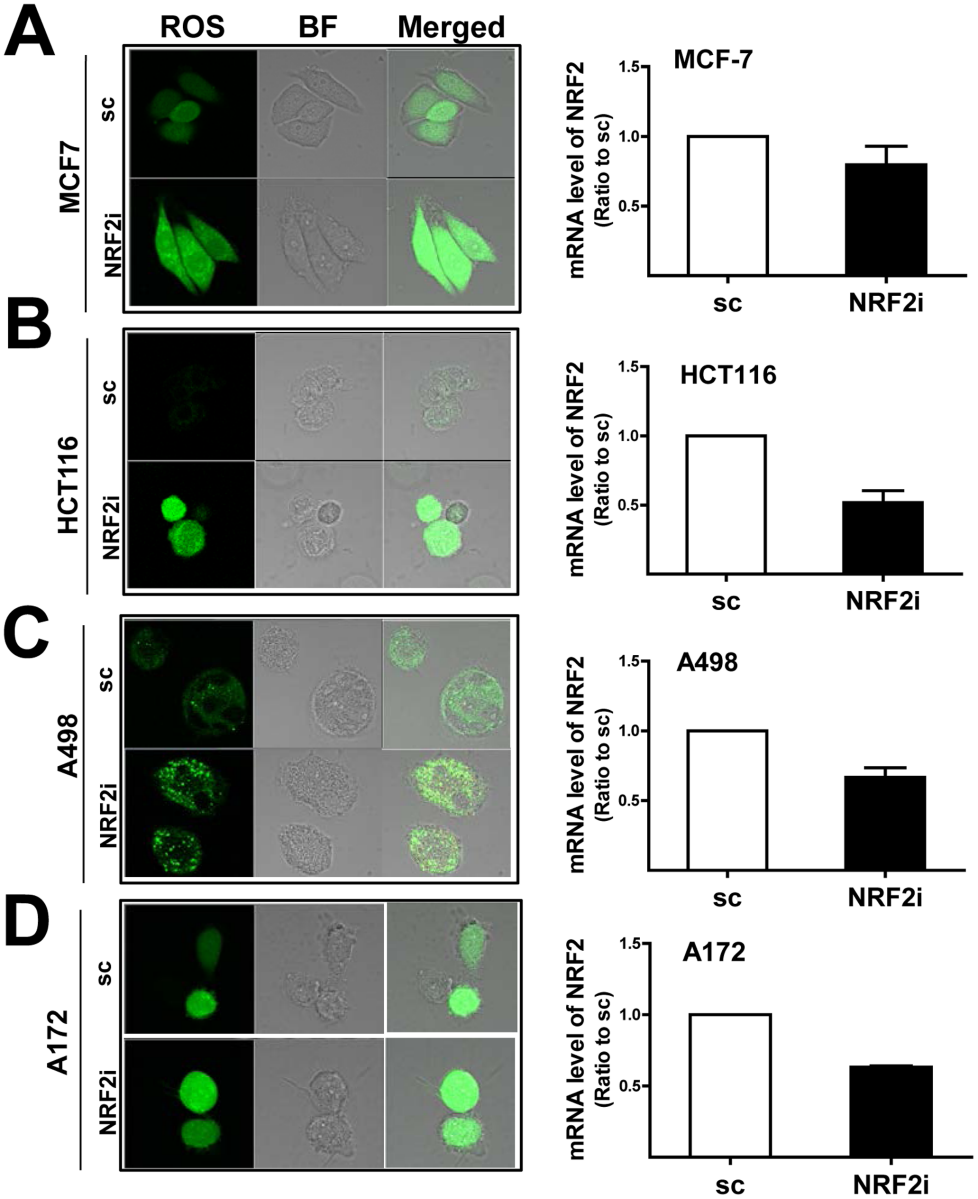
The combination of *NRF2* shRNA delivery and Pba PDT elevates ROS levels in additional cancer cell lines. The breast carcinoma MCF7 cell line (A), colon carcinoma HCT116 cell line (B), renal carcinoma A498 cell line (C), and glioblastoma A172 cell line (D) were transiently incubated with lentiviral particles containing *NRF2* shRNA. Cells were treated with PDT, and the levels of cellular ROS were monitored with DCFDA staining. Transcript levels of NRF2 were measured using relative quantification of real-time PCR in each cell line. BF, bright field; ×100 magnification.

Next, we examined the efficacy of *NRF2* siRNA delivery in PDT sensitization of 4 additional types of cancer cells: human breast carcinoma cell line MCF7, colon carcinoma cell line HCT116, renal carcinoma cell line A498, and glioblastoma cell line A172. These cells were incubated for 48 h with the *NRF2* shRNA-containing lentiviral particle and then subjected to PDT. Subsequently, cellular ROS level was assessed as a marker of PDT efficacy. The level of knockdown was not as high as in the stable cell line. Nevertheless, lentiviral delivery of shRNA significantly reduced the level of *NRF2* mRNA in each cell line: 44% reduction in HCT116, 35% reduction in MCF7, 22% reduction in A498, and 38% reduction in A172 (Fig. 9A–9D). Under conditions of *NRF2* depletion, PDT-induced ROS was substantially increased in all cell types. These data confirmed the beneficial effects of *NRF2* siRNA for sensitizing cancer cells to PDT.

## Discussion

The activation of NRF2 using pharmacological interventions or genetic approaches can prevent oxidative stress-associated diseases, such as inflammatory diseases and cancer [27], [28], [31]. Whereas, a constitutive increase in NRF2 expression can promote cancer cell survival in the stressful tumor environment and in the presence of chemotherapeutic agents [33], [41], [42]. In the present study, we investigated NRF2 as a potential molecular determinant of the efficacy of Pba-based PDT in human breast carcinoma MDA-MB-231 cells and colon carcinoma HT29 cells. Stable knockdown of *NRF2* in both cancer cell lines enhanced cell death and substantially increased ROS levels following Pba-PDT as compared to the respective control cell lines. In particular, the level of singlet oxygen, which is a direct product of Pba photoactivation, was significantly higher in *NRF2*-knockdown cancer cells than in control cells, suggesting differential levels of cellular Pba between these cell lines. Indeed, confocal microscopic observation of fluorogenic Pba showed significantly higher cellular accumulation of Pba in knockdown cells as compared to control cells. A subsequent analysis of the expression of membrane drug transporters revealed that the basal expression of *BCRP* is NRF2-dependent. Among the measured ABC transporters and SLC transporters, only BCRP was down-regulated by *NRF2* knockdown. Accordingly, treatment with a BCRP-specific inhibitor enhanced PDT sensitivity in control cells but not knockdown cells. These results indicate that the inhibition of *NRF2* in cancer cells could enhance sensitivity to Pba-PDT by decreasing *BCRP* expression. In addition to photosensitizer accumulation, *NRF2* knockdown affected the expression of mitochondrial ROS-counteracting proteins. Among the examined ROS-scavenging proteins, including SOD1/2, CAT, GPX1/4, PRDX3/5, and TXN2, the transcript level of *PRDX3*, which is a major ROS scavenger in the mitochondria, was down-regulated by *NRF2* knockdown in MDA-MB-231 cells. Finally, we demonstrated that PDT-induced ROS was substantially elevated by the delivery of shRNA to other types of human cancer cell lines, including breast carcinoma MCF-7, colon carcinoma HCT116, renal carcinoma A498, and glioblastoma A172 cells. These results confirm that NRF2 is a molecular determinant of Pba-PDT sensitivity in various types of cancer cells.

BCRP, an ABC transporter, exists in the plasma membrane as a homodimer, which is formed through disulfide bonds between extracellular cysteine residues of the BCRP molecule [43]. BCRP is overexpressed in many types of cancer and is strongly associated with chemoresistance by mediating the efflux of a wide range of anti-cancer agents, such as anthracyclines and methotrexate [21], [44], [45]. Chlorophyll-derived dietary phototoxins are known substrates of BCRP in animals. Using *bcrp-*deficient mice, Jonker et al. demonstrated that Pba-induced skin phototoxicity was increased in the absence of BCRP [24]. Later, Robey et al. provided direct evidence that Pba is a specific substrate of BCRP [23]. Among cancer cell lines expressing high levels of multidrug resistance-associated protein-1, P-glycoproteins, or BCRP, Pba transport was only detected in cells expressing BCRP and this transport was inhibited by a BCRP-specific inhibitor. Furthermore, other type of protoporphyrins, such as the clinical photosensitizer photochlor and verteporfin, are also known as BCRP substrates [46]. In a retrospective examination of BCRP levels in clinical samples, BCRP expression was significantly associated with the efficacy of photofrin-PDT in patients with early lung cancer [47]. Therefore, BCRP inhibition may be an effective method for enhancing PDT efficacy. Indeed, treatment of cells with imatinib mesylate, a BCRP inhibiting tyrosine kinase inhibitor, increased levels of the photosensitizer within tumors and enhanced the efficacy of PDT in mice [46]. These data implicated the role of BCRP in regulating the efficacy of PDT.

Drug transporters are transcriptionally regulated: the aryl hydrocarbon receptor (AhR), pregnane X receptor (PXR), and constitutive androstane receptor (CAR) are involved in their regulation [48], [49]. Additionally, several research groups have reported the association of NRF2 in regulating the expression of drug transporters. Previously, Hayashi et al. reported that *Mrp-1* expression is up-regulated by treatment with diethyl maleate, which activates NRF2, and that the basal expression of Mrp-1 was lower in *nrf2*-deficient embryonic fibroblasts than that in wild-type cells [50]. Maher et al. demonstrated that the administration of NRF2 activators in mice increased the expression of Mrp2-6 in the liver [51]. Later, BCRP expression was reportedly up-regulated upon treatment with *tert*-butylhydroquinone through NRF2 in HepG2 cells [52]. In our study, NRF2 affected basal expression of BCRP. Specifically, the mRNA level and protein levels of both the BCRP monomer (70 kDa) and homodimer (140 kDa) were significantly decreased by *NRF2*-knockdown. In contrast, the levels of *MRP1-6*, *MDR1*, and *OCTN1-2* were unaffected by *NRF2-*knockdown, indicating that basal expression of BCRP is highly dependent on NRF2. Based on this specific link between NRF2 and BCRP in cancer cells, together with the effect on mitochondrial antioxidant genes, NRF2 can be a novel molecular target for enhancing the efficacy of Pha-PDT. This principle was validated in other cancer cell types, including HT29, HCT116, MCF-7, A498, and A172 cell lines.

The cellular mechanism underlying PDT phototoxicity involves apoptosis and necrosis [6]. As a mitochondria-dependent pathway, it was shown that PDT-ROS induces inner mitochondrial membrane permeabilization and subsequent cytochrome c release, which leads to apoptotic cell death [40], [53]. In a confocal microscopic determination, we observed that cellular Pba is primarily co-localized with the mitochondrial dye, and this implies that mitochondria-mediated apoptosis can be involved in PDT cytotoxicity in MDA-MB cells. However, the treatment with pan-caspase inhibitor could not completely block PDT-mediated cell death. This indicates that PDT-induced cell death is developed by a complex mechanism. Another mechanism underlying PDT phototoxicity is necrosis. Although necrosis is a passive mechanism, necroptosis or programmed necrosis has recently emerged as a novel mechanism of cell death [54], [55]. Oxidative stress has been proposed as a stimulator of necroptotic cell death [56]. Mouse embryonic fibroblasts with the deletion of *RIPK* gene, an essential component in necroptotic response, were resistant to hydrogen peroxide-induced cell death [57]. Treatment of mouse hippocampal cells with necroptosis-specific inhibitor necrostatin-1 prevented GSH depletion-induced cytotoxicity [58]. In our study, most of PDT-exposed *NRF2* knockdown cells were Annexin+/PI- and Annexin+/PI+ populations, which represent the early apoptotic and necrotic/late apoptotic cells, respectively. Accordantly, PDT-induced knockdown cell death affected by pharmacological inhibitors of apoptosis and necroptosis. The co-incubation of NRF2i cells with either Z-VAD-FMK (pan-caspase inhibitor) or necrostatin-1 attenuated PDT-induced cell death with a similar degree. These results indicate that Pba-PDT can induce cell death through both apoptotic and necroptotic mechanisms.

The role of NRF2 in cancer has received increasing attention, because constitutive activation of NRF2 has been identified in many human cancers [33], [41]. Several different mechanisms have been proposed for the constitutive activation of NRF2 [42]. First, somatic mutations on *KEAP1* or *NRF2* have been reported to cause of gain-of-function NRF2 in cancer cell lines and tumor tissues. Second, reduced *KEAP1* expression due to promoter hypermethylation has been observed. Third, the *NRF2* transcription can be increased by signaling through oncogenes, such as *c-Myc* and *K-Ras*. Fourth, accumulation of p21 or p62 can disrupt the binding of KEAP1 to NRF2. Fifth, certain cancer metabolites, such as fumarate, can modify KEAP1 protein, causing it to activate NRF2. Although the underlying mechanisms may differ, the ultimate activation of NRF2's target genes, including detoxifying enzymes, antioxidant proteins, and drug transporters, confers cancer resistance to anti-cancer drugs. Therefore, the inhibition of *NRF2* expression using interfering RNA could restore chemosensitivity in various cancer cells [59], [60]. Thus the potential role of NRF2 in PDT response can be hypothesized. It has been demonstrated that the expression of *HO-1*, a target gene of NRF2, is involved in the protection against photofrin-PDT toxicity and that HO-1 inhibitors potentiate the anti-tumor effect of PDT [37]. Another group reported that PDT with Pba, delta-aminolevulinic acid, and protoporphyrin IX increased the expression of *BCRP* and *HO-1* expression [38]. In that study, the authors raised the possibility that individual differences in PDT response can be associated with activation of BCRP and HO-1 in tumors. Together with these findings, our results provide clear evidence that NRF2 inhibition sensitizes cancer cells to Pba-based PDT through the modulation of BCRP expression.

Collectively, our results suggest that NRF2 is a molecular determinant of the efficacy of PDT in various cancer cell types. Therefore, manipulation of NRF2 activity in tumors can be a novel strategy for enhancing the efficacy of PDT.
